# Supplementary material for: Controlling microbial co-culture based on substrate pulsing can lead to stability through differential fitness advantages
Source: PLoS Comput Biol. 2022 Oct 31;18(10):e1010674. doi: 10.1371/journal.pcbi.1010674 (PMC9648842; doi:10.1371/journal.pcbi.1010674)
Supplement: S2 File — File containing a MONCKS usage and workflow description. (PDF) [file pcbi.1010674.s002.pdf]

Controlling microbial co-culture populations based on substrate pulsing can lead to stability through differential fitness advantages.

J.Andres Martinez[1], Matheo Delvenne[1,◑], Lucas Henrion[1,◑], Fabian Moreno[1,◑], Samuel Telek[1], Christian Dusny[2], Frank Delvigne[1,*]

1 TERRA Research and Teaching Centre, Microbial Processes and Interactions (MiPI), Gembloux Agro-Bio Tech, University of Liége, Gembloux, Belgium.
2 Microbial Single Cell Analysis, Department of Solar Materials, Helmholtz-Centre for Environmental Research- UFZ Leipzig, Permoserstr. 15 04318 Leipzig, Germany.

* f.delvigne@uliege.be

Supplementary File 2: Simplified MONKS simulation workflow

The MONod-type Co-culture Kinetic Simulation toolbox (MONCKS) is part of the MiPI Bioprocess Modeling and Simulation toolbox (mBMStoolbox). The former toolbox starts by taking the cybernetic model parameters for two strains, i.e., *E. coli* and *S. cerevisiae*, and using them in a numerical integration calculation with a small Δt (i.e., 0.01 h) to calculate their interaction with a common external metabolite pool which simulates the environmental conditions of the bioreactor. Each Δt, it is possible to add a flux of metabolites to simulate a constant flow or pulses. MONCKS use in the present work was comprised of a series of functions that allow parametrizing the initial conditions of the bioprocess (`BMS_MONCKS_pBioreactor_xlsx`), read and parametrize two strains forming the co-culture system (`BMS_MONCKS_rModels`), perform the numerical simulation of co-culture growth during the bioprocess (`BMS_MONCKS_cSimulation`), read Experimental data sets (`BMS_MONCKS_rExpData_mat`) and compare the fraction of events experimental data to the simulations to assess the simulation quality (`BMS_MONCKS_qSimulation_Xfrac`). The simplified example pipeline in MATLAB can be written as follows:

```
%%%␣MONCKS␣Pipeline␣example␣for␣the␣S.␣cerevisiae␣and␣E.coli␣co-culture␣used␣in␣this␣work
[BMS_BioPar]=BMS_MONCKS_pBioreactor_xlsx('MONCKS_HiFreq.xlsx');
%%%␣File␣in␣brackets␣can␣be␣changed␣for,i.e.,'␣MONCKS_LowFreq.xlsx',␣'MONCKS_Cont.xlsx'
[BMS_AModel,BMS_BModel,BMS_BioPar]=BMS_MONCKS_rModels(BMS_BioPar);
[BMS_BioData,BMS_ASimData,BMS_BSimData]=BMS_MONCKS_cSimulation(BMS_AModel,BMS_BModel,BMS_BioPar);
[BMS_ExpPar,BMS_ExpData]=BMS_MONCKS_rExpData_mat('MONCKSExpDataHifreq');
%%%␣File␣in␣brackets␣can␣be␣changed␣for,i.e.,'␣MONCKS_LowFreq.xlsx',␣'MONCKS_Cont.xlsx'
[BMS_BioPar,BMS_QlyData]=BMS_MONCKS_qSimulation_Xfrac(BMS_BioPar,BMS_BioData,BMS_ExpData);
```

The simulation pipeline starts by reading a .xlsx file with a tab named `Bioreactor` containg the initial fermentation data (Fig. ??). Where `BIOa` and `BIOb` refers to the first and second `.mat` cybernetic model parameter files, respectively. `To`, `Tf` and `dt` are the initial, final and delta times, respectively. `Feed type` can be set in "Discontinuous" and "Continuous" modes, referring to pulses on Feeding times or pulses on Feed concentrations, respectively. `Feed start` is in hours, `Dilution rate` in 1/h, and the `Volume` in L. the `Feed function` used in this work was a "square" wave function, and the `Period Frequency` and `On Time Fraction` are set for simulation in this file. The final part of this file contains the vectors of concentrations for the diverse metabolites, including the starting biomass of both microorganisms, for the Feed and the Initial Concentration. Examples on this files can be found at https://gitlab.uliege.be/mipi/published-software/mipi-model-and-simulation-database.

After reading the initial bioprocess file, the pipeline continues with the reading of the model parameters for the defined microorganisms. These files are `.mat` files containing the structure `BMS_Model`, which includes the main parameters for the cybernetic simulation of the behavior of a particular strain.

After acquiring the model parameters for each strain, the pipeline proceeds to numerically simulate the co-culture and the bioreactor environmental conditions according to the simplified algorithm shown in Figure 1.

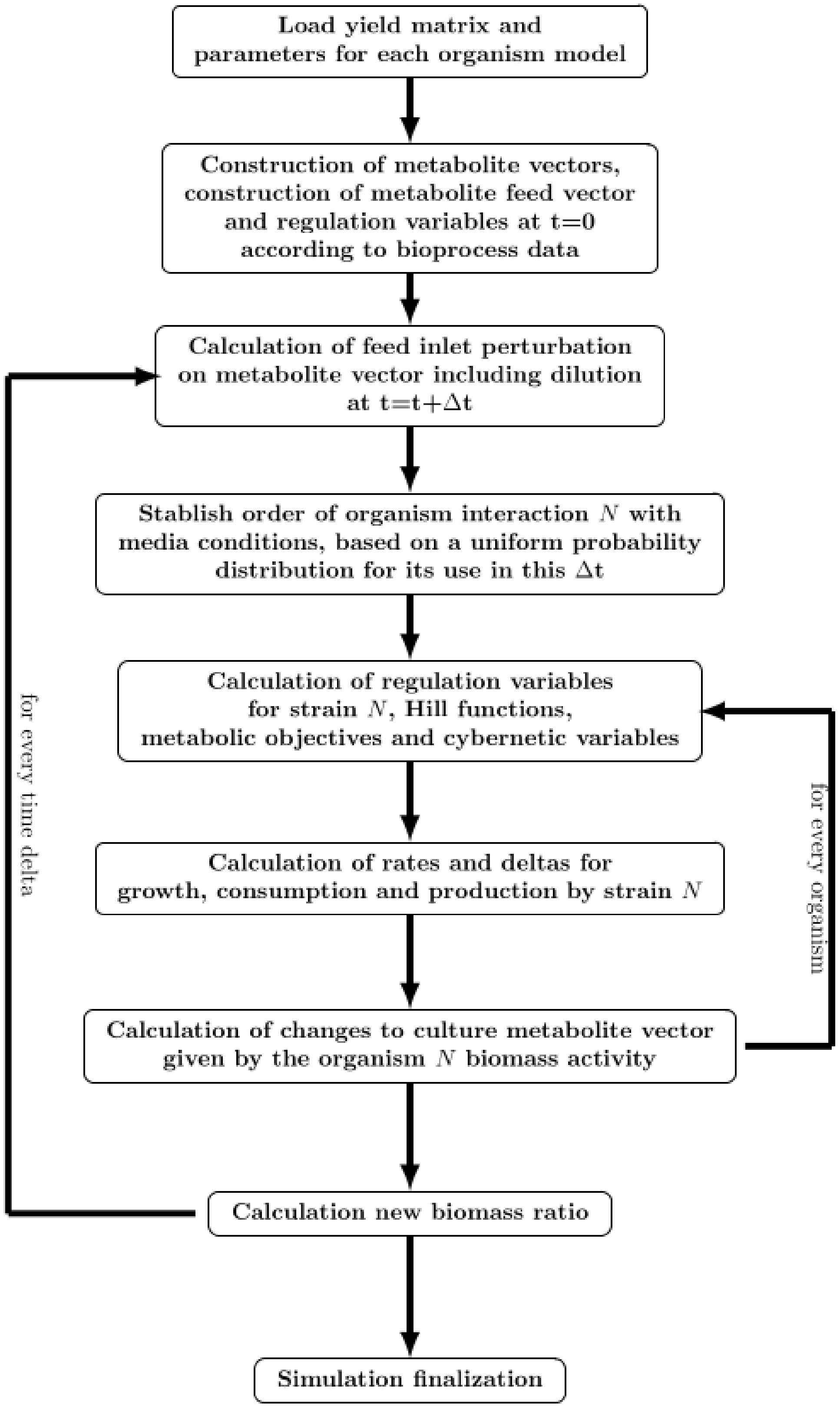

Fig 1. Simplified algorithm steps diagram used in MONCKS framework used for simulating the co-culture experiments

Finally, the pipeline reads and compares selected experimental data to the. In this case, we used the fraction of events as the quality reference for the modeling approximation. All the functions and data sets can be found in the repositories at: https://gitlab.uliege.be/mipi/published-software/mbms-toolbox, used databases can be found at: https://gitlab.uliege.be/mipi/published-software/mipi-model-and-simulation-database.
