# Supplementary material for: Controlling microbial co-culture based on substrate pulsing can lead to stability through differential fitness advantages
Source: PLoS Comput Biol. 2022 Oct 31;18(10):e1010674. doi: 10.1371/journal.pcbi.1010674 (PMC9648842; doi:10.1371/journal.pcbi.1010674)
Supplement: S4 File — File containing extended data for the metabolite experimental data and model simulations for continuous and the high- and low-frequency feeding regimes. Extended figures related to the S. cerevisiae relative enzyme for monoculture and co-culture at low-frequency feeding regimes. (PDF) [file pcbi.1010674.s004.pdf]

Controlling microbial co-culture populations based on substrate pulsing can lead to stability through differential fitness advantages.

J.Andres Martinez[1], Matheo Delvenne[1,Y], Lucas Henrion[1,Y], Fabian Moreno[1,Y], Samuel Telek[1], Christian Dusny[2], Frank Delvigne[1,*]

1 TERRA Research and Teaching Centre, Microbial Processes and Interactions (MiPI), Gembloux Agro-Bio Tech, University of Liége, Gembloux, Belgium.
2 Microbial Single Cell Analysis, Department of Solar Materials, Helmholtz-Centre for Environmental Research- UFZ Leipzig, Permoserstr. 15 04318 Leipzig, Germany.

* f.delvigne@uliege.be

Supplementary File 4: Continuous and Discontinuous culture supplementary figures

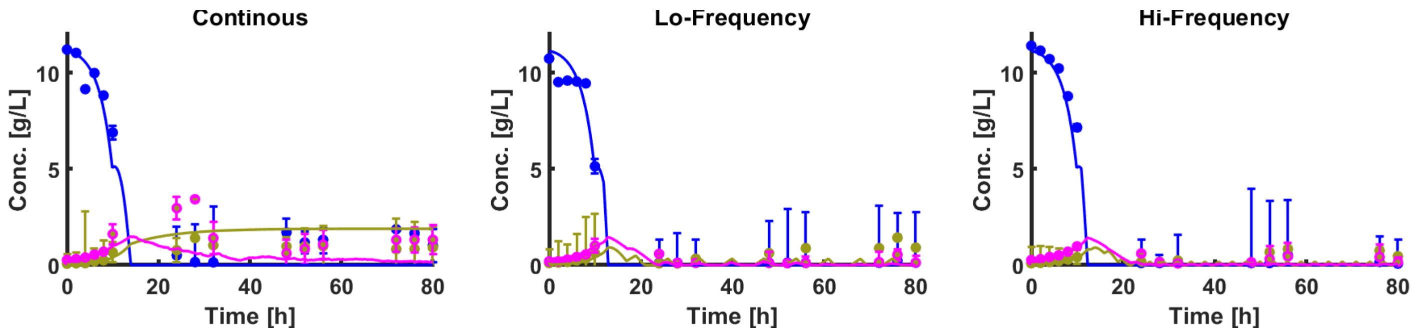

Fig 1. Measurement data set coverage for the Continuous, Low-frequency and High-frequency feed co-culture experiments from time 24 to 80.

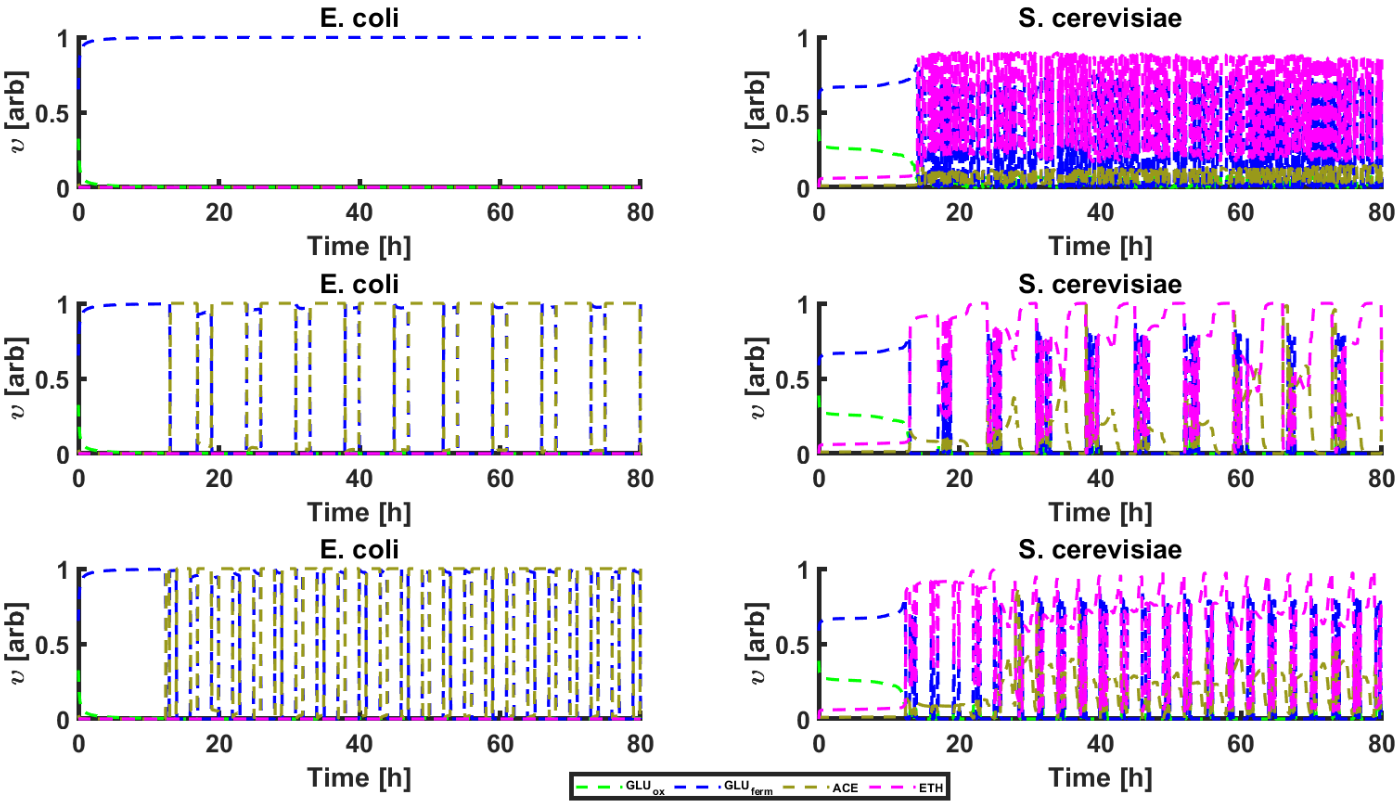

Fig 2. Cybernetic variable υ for *E. coli* and *S. cerevisiae* during the simulations made for Continuous culture (up), low frequency (middle) and high frequency (down) pulsing experiments.

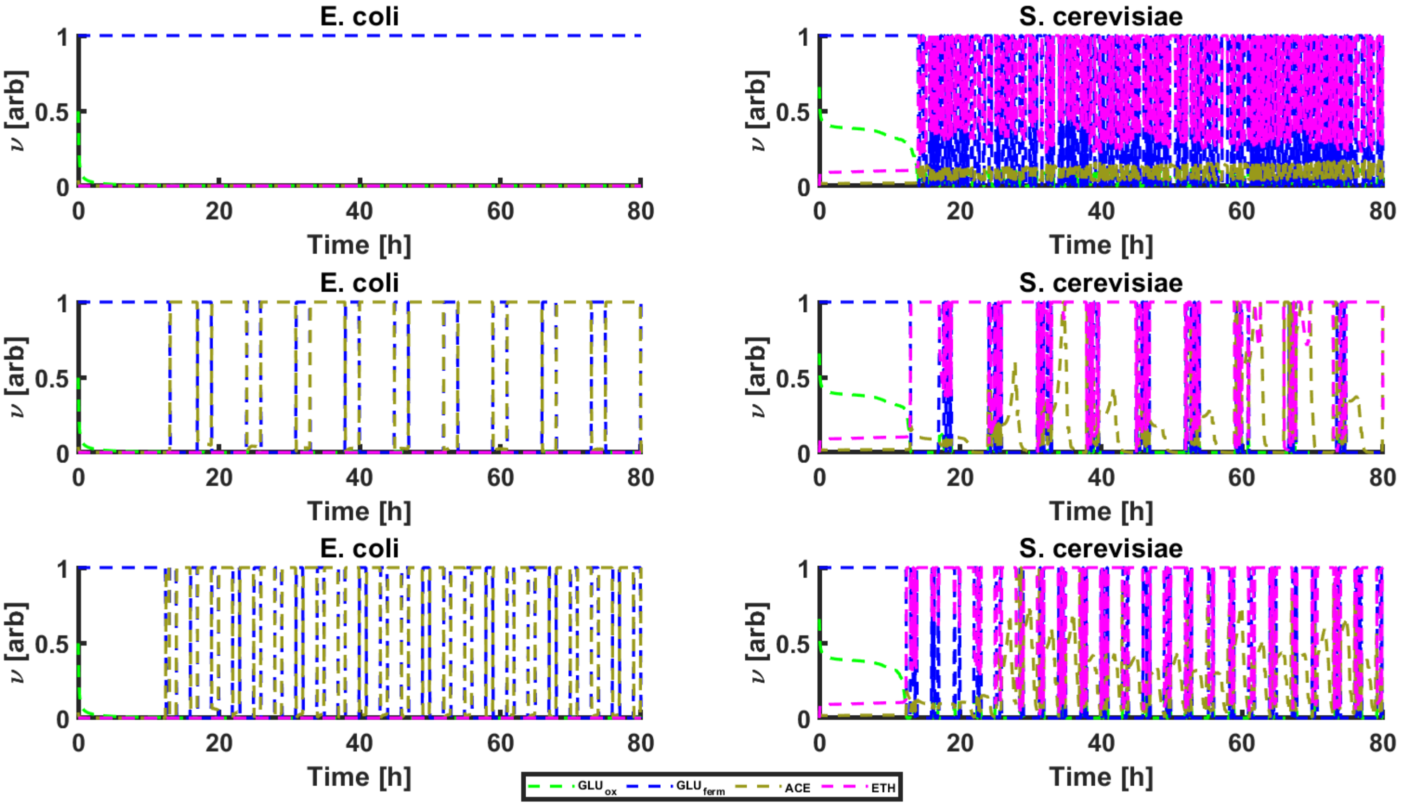

Fig 3. Cybernetic variable ν for *E. coli* and *S. cerevisiae* during the simulations made for Continuous culture (up), low frequency (middle) and high frequency (down) pulsing experiments.

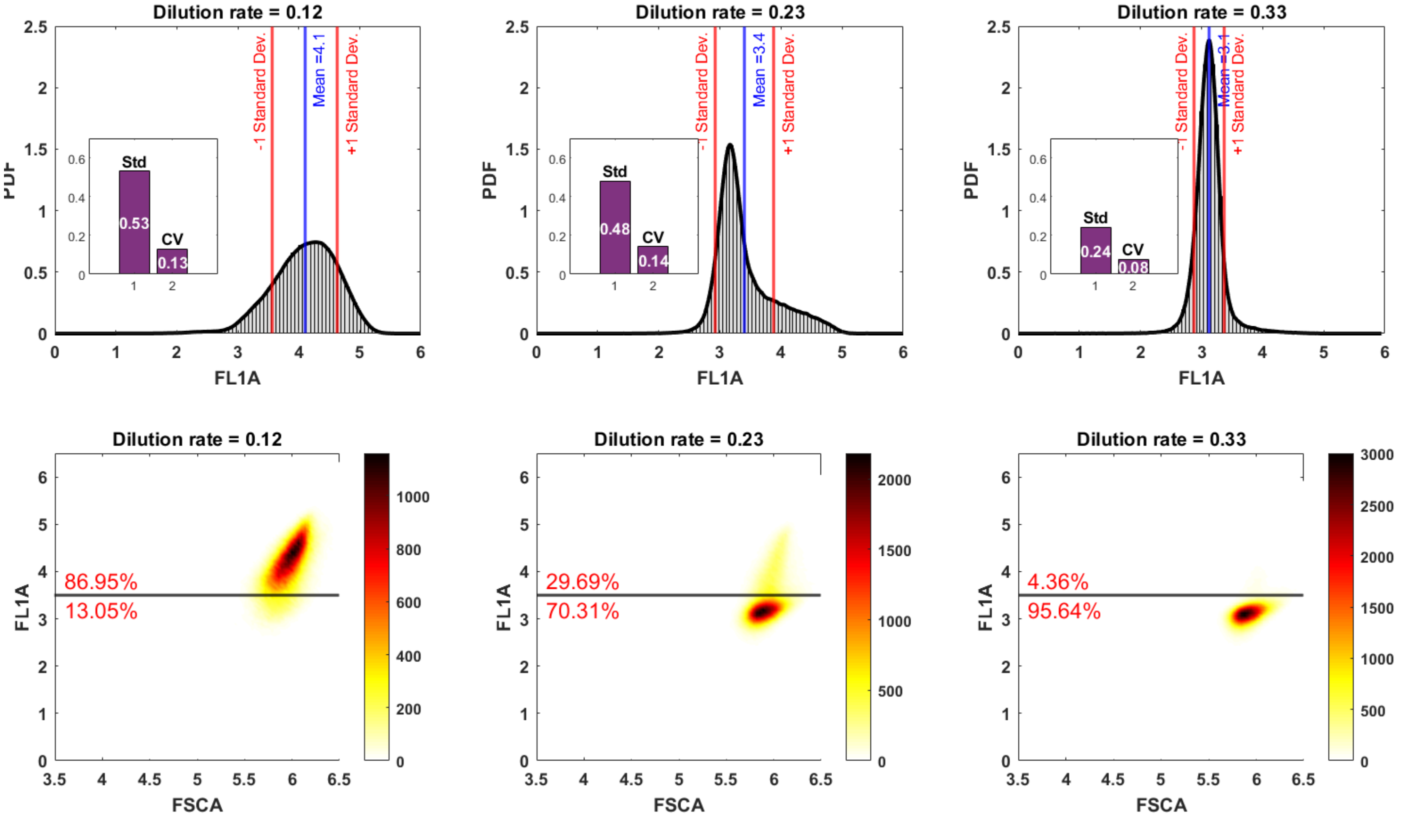

Fig 4. top row: FSCA Probability density function, mean, standard deviations and coefficient of variation for the axenic continuous cultures of *S. cerevisiae* at dilution rates of 0.12, 0.23 and 0.33 h^{-1}, respectively. bottom row: Flow citometry data for the triplicates at the different dilution rates and their GFP^{+} and GFP^{-} percentual calculations along the threshold at 3.5.

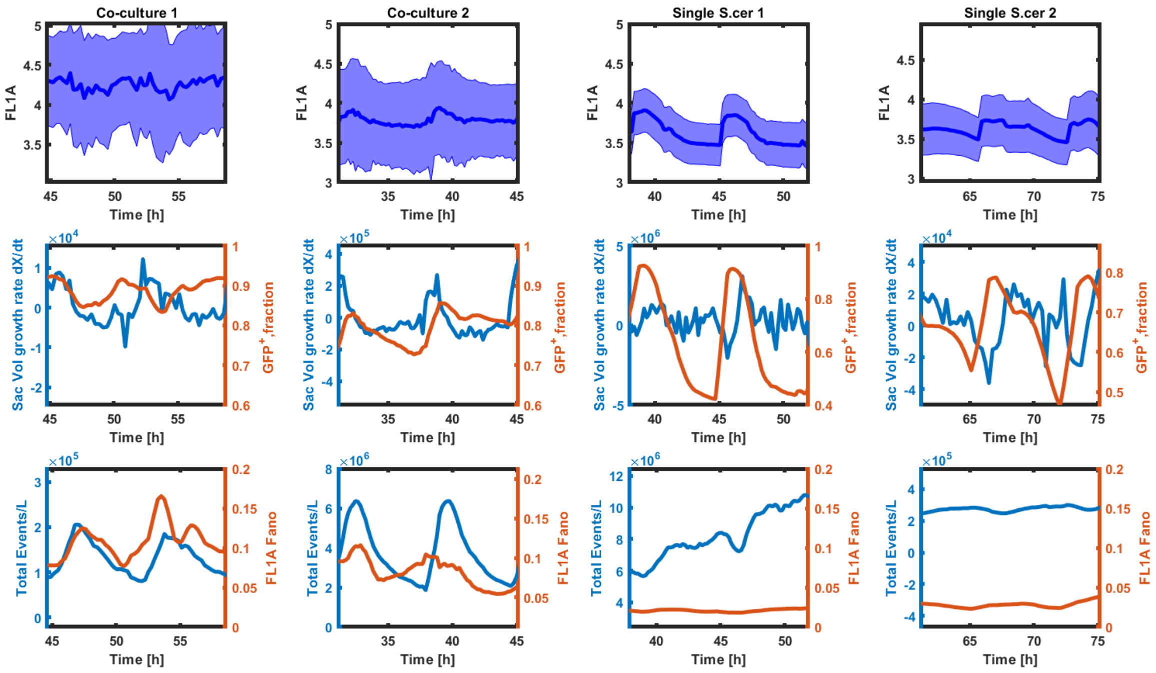

Fig 5. Time evolution of FL1, approximated growth rate, GFP^{+} fraction, total events over liter and Fano factor across example cycles for the low-frequency feed regime experiments. Plotted values correspond for *S. cerevisiae* population in coculture and in single culture (duplicates)
