# Supplementary material for: Controlling microbial co-culture based on substrate pulsing can lead to stability through differential fitness advantages
Source: PLoS Comput Biol. 2022 Oct 31;18(10):e1010674. doi: 10.1371/journal.pcbi.1010674 (PMC9648842; doi:10.1371/journal.pcbi.1010674)
Supplement: S5 File — List of abbreviations used in this work. (PDF) [file pcbi.1010674.s005.pdf]

Controlling microbial co-culture populations based on substrate pulsing can lead to stability through differential fitness advantages.

J.Andres Martinez[1], Matheo Delvenne[1,Y], Lucas Henrion[1,Y], Fabian Moreno[1,Y], Samuel Telek[1], Christian Dusny[2], Frank Delvigne[1,*]

1 TERRA Research and Teaching Centre, Microbial Processes and Interactions (MiPI), Gembloux Agro-Bio Tech, University of Liége, Gembloux, Belgium.

2 Microbial Single Cell Analysis, Department of Solar Materials, Helmholtz-Centre for Environmental Research- UFZ Leipzig, Permoserstr. 15 04318 Leipzig, Germany.

* f.delvigne@uliege.be

Supplementary File 5: Nomenclature

S	Substrate
GLU	Glucose
ACE	Acetate
ETH	Ethanol
Ψ	Virtual enzyme
X	Biomass
Ψ_σ	Virtual enzyme related to the S consumption
r_σ	Consumption rate of S
A to N	Metabolites
a to n	Subindex referring to the Metabolites A to N
α to η	Subindex referring to the Metabolites A to N as substrates
M	Extracellular metabolite pool
Y	Yields
ε^c	Constitutive enzyme expression rate
ε^i	Inducible enzyme expression rate
δ	Enzymatic degradation rate
μ	Growth rate
K	Monod-type affinity constant
q_σ	Consumption rate constant of S
$\Psi\sigma_{rel}$	Relative enzyme concentration

H_σ	Monod-type hill function for uptake rate of S
Φ	Global metabolic state
υ	Cybernetic variables regulating expression
ν	Cybernetic variables regulating activity
P	Physiological behaviour on the cultivation conditions
D	Dilution rate
w	Frequency of pulsing
s	Time fraction for feed pulse
