# Supplementary material for: Controlling microbial co-culture based on substrate pulsing can lead to stability through differential fitness advantages
Source: PLoS Comput Biol. 2022 Oct 31;18(10):e1010674. doi: 10.1371/journal.pcbi.1010674 (PMC9648842; doi:10.1371/journal.pcbi.1010674)
Supplement: Attachment — Submitted filename: Reviewers (2).docx [file pcbi.1010674.s006.docx]

Dr. Sunlil Laxman and Dr. Mark Alber	September 21^st^,2022

Guest and Second editors, PLOS Computational Biology

Dear editors,

We very much thank the comments made on the manuscript and the effort made by you and the reviewers that have helped us to enhance the quality and content of our work. We hope that with the modifications done to the original manuscript and the answers described below, we will satisfice the expectation you and the reviewers have for our work. We will now address the considerations made by each reviewer individually and make some finals remarks afterwards.

**Reviewer #1:** In the present manuscript Martinez et al utilized a cybernetic model to study the co-culture of two classic model organisms, Escherichia coli and the Saccharomyces cerevisiae. The authors acquired extensive experimental data first by growing the microbes in monoculture and subsequently in co-culture with different glucose pulses. These experiments offer a promising proof of concept for their simulations as well as reveal additional information on the social interactions of these microbes. The described workflow is interesting and holds value for numerous applications. However, there are some open issues and addressing them will improve the clarity of their findings.

Major comments

1. Line 294 “The beginning of the culture and remains as such as long as the GLU concentration is non-limiting (> 1.5 g/L).”. Could the authors justify how they selected this value? They do not reference any of their figures and when Fig. 2A is carefully considered growth of E. coli seems limited with 2.5 g/L of glucose and severely impaired even with 5 g/L.
   a. *We have changed the Line now 292 sentence to: “…the beginning of the culture and remains as such if the GLU is non-limiting (Figure 3 A and B). Between the range of initial concentration tested (20 to 1.25 g/L), the maximum growth rate achieved by E. coli is reduced by only 12.4% (Figure 3 B), while the model Ks was found to be near 0.11 g/L, where we expect a 50% growth rate decrease. The reduction observed in Figure 3 B on growth rate was found on times with GLU at least below to approx. 0.5g/L, where this substrate limitation becomes stringent (Figures 2 and 3 B).*
2. Line 506 “Most of these systems are based on cross-feeding and quorum sensing modules”. In this part of the discussion the authors aim to separate their approach from other approaches based on cross-feeding. However, their co-culture remains stable based on the consumption of “metabolic by-products” like acetate. I would argue that S. cerevisiae taking up and consuming the acetate that was produced from E. coli is a form of metabolite cross-feeding as described in Smith et al. 2019 (https://doi.org/10.3389/fevo.2019.00153). Could the authors elaborate how they believe that their tool is distinct from other modelling cross-feeding based approaches?
   a. *Thanks for your comments. Regarding the sentence of Line 506 (Now 531), it was mostly related to the design of synthetic genetic parts dedicated to maintaining species balance during cultivation. We recognize the lack of clarity; therefore, we have changed the sentence to: “Most of these controlled co-cultivation systems are constructed based on cross-feeding and quorum sensing modules…”. Regarding the second part of your comment, indeed, the acetate consumed by S. cerevisiae produced by E. coli is a form of cross-feeding. To better discuss the differences between other cross-feeding approaches, we added the following sentences and the reference you kindly provided us on line 533: “However, several types of cross-feeding (e.g., Substrate, metabolite, mutual, augmented cross-feeds) can be found even between wild type organisms, an thus defining their social interactions [60]. In this work, we have focused our effort on the stabilization of wild-type strains of E.coli and S.cerevisiae considering substrate competition and possible overflow metabolite cross-feeding”.*
3. Line 554 “The only requirement for the application of MONCS to the simulation of microbial co-culture is the determination of the main metabolic pathways involved…”. In this study the authors chose very well characterised model organisms and still had to contact long and sophisticated experiments in mono-culture prior to simulating the co-cultures. Could they describe an example how they envision use of their tool, as this above described statement seems like an oversimplification for the required model input.
   a. *Thanks for this very important input. To address it, we added the following sentences in line (582):” This metabolic pathway determination can be performed by product yield characterization on single substrate growth experiments or by yield analysis from stoichiometric matrices reconstructed from partial (e.g., core-metabolism) or genome-scale annotations [24,30,31,70,71].”.*

Minor comments

1. For figure 2, inclusion of the legend and the explanation of the symbols and colours on the figure and not only on the legend, would increase the clarity of the whole figure.

2. Figure 6 i(especially 6B) is illegible fact that limits the comprehension of the depicted data.

3. Clarity of Figure 9A and 9C would improve with inclusion of a grade scale for the colours.

a. *Thank you for your comments. We made changes to figures considering all reviewer’s comments on them.*

**Reviewer #2:** The manuscript is much improved – I found it very easy to follow the experimental design and the authors did a good job of connecting each section of the results. I have a couple of comments that I am sure will be easy for the authors to address.

The authors use biomass as the measure of the amount of each of the species. An important difference between E. coli and Yeast is their size difference, with yeast being ~10 times bigger than E. coli. This is important when taking dilution into account for experiments- not be a problem for the range that the authors show in figure 6 but perhaps they could note that for extreme dilutions, say, in the range of 0.001 or more, it may be that Yeast is in more danger of being diluted from the culture (and therefore, to go extinct) than E. coli. Did the authors explore the greater level of dilution and its effect on the co-culture? The authors may want to briefly mention what the effect of a greater dilution would be since one might assume from those simulations that more dilution would lead to more stability.

a. *Thanks for your observation. Since in the figure we are considering dilution rates, the growth and the outflow of the organism depend on the mass concentrations and not on the number of individuals. However, it is true that at some dilution rate limits, organisms are more probable to be extinct, depending specifically on their growth rates. In our case study, the maximum growth rate calculated for E.coli is around 0.4 h-1 while for S.cerevisiae is around 0.3 h-1. Therefore, having dilution rates above this latter value would inevitably result in a washout and extinction of the yeast in the culture.*

The figures look really great, and the authors should be commended for putting together such clear illustrations that also help the reader understand the manuscript. It would be helpful if the font sizes for figures 5, 6, and 8, etc, were standardized to a larger size since they are currently difficult to read.

a. *Thank you very much for your kind observations. We made changes to figures, considering all reviewer’s comments on them.*

**Reviewer #3:** In the manuscript titled ‘Controlling microbial co-culture based on substrate pulsing can lead to stability through differential fitness advantages’, the authors develop a cybernetic model to understand the dynamics in microbial co-culture. To illustrate this, the authors perform the experiments and simulations using two model organisms S. cerevisiae and E. coli. While these organisms and their cocultures are very well-studied in literature, the authors here seek use cybernetic modelling to demonstrate its’ applicability. Overall, I find the work quite interesting, however have a lot of concerns that need attention before the manuscript can be considered for publication. I have divided my comments based on different sections.

Materials and methods [Main text and Supplementary Files]

Please explain the meaning of all the variables that have been used in different equations. It is very hard to interpret without them.

a. *Thanks for the observation. We have added a Nomenclature box to increase the readability of equations.*

Cybernetic modeling reveals differences in metabolic fitness upon the diauxic shift in mono-cultures

The authors seem to use monoculture values to model coculture.

Since the authors’ main theme is to perform cocultures, I’m not too sure if the parameters obtained from monocultures can be used for coculture modelling.

The authors also do not include an interaction term between E. coli and S. cerevisiae in the cybernetic model, which would have effects on the predictions [as authors themselves recognize in Lines 392-398]. One possible way to account for this might be to do a coculture experiment and determine the parameters to fit into the model.

a. *Thank you for your remarks and recommendation. For all our models, single and co-culture, the same parameters were used for the modelling. We would not expect the main parameters, such as the maximum growth rate, affinity constants, or yields concerning each substrate, to change during continuous culture or even in the co-culture because the metabolic capabilities of each strain define them. However, the dynamic behaviour and interplay between the different metabolic capabilities are expected to change depending on the environment and other microorganisms’ effects on the latter. This is precisely the virtue of using cybernetic modelling approaches in the mathematical description of this phenomena since the cybernetic sections of the model tune to the information the dynamic changes in the environment to the metabolic behaviour of a strain towards a metabolic objective, which in this case is growth. As presented in the manuscript, this modelling approach allowed us, with enough accuracy, to render some simple but critical interactions between the two strains, which are the competition for environmental resources, and the potential cross-feeding resulting from overflow metabolisms.*

*It is true that, regarding the co-culture models, more complexity can be added to end with a closer rendering of the behaviour of the microorganism during the culture. However, two main obstacles are the increasing numbers of parameters needed to address the increasing complexity, which would need more extensive insights and data into the interaction mechanisms (e.g., production of toxic compounds, functions to increased death rate constants, production of scaffolds for biofilm formation, diffusion coefficients around them, among many others) and their specificity regarding each pair of microorganisms that would be wished to model. The presented modelling framework can indeed be used to include this type of phenomenon; however, more effort needs to be then done into constructing the individual functions and parameters describing them, along with more detailed and define datasets. Despite this, in the current state, the presented modelling approach successfully describes and predicts some relevant systemic characteristics that allowed us to find critical setpoints for the control and design of more stable co-cultures.*

Fitness disparity between metabolic phenotypes leads to population imbalances during continuous cultures with constant environmental conditions

2. Does ethanol production by S. cerevisiae inhibit the growth of E. coli? Was this factor included in the model?.
   a. *Ethanol is commonly considered a growth inhibitor for several microorganisms. In the case of E. coli, on the Sawada and Nakamura studies on the growth inhibitory and lethal effects of ethanol on Escherichia coli (*[*https://doi.org/10.1002/bit.260290611*](https://doi.org/10.1002/bit.260290611)*), it can be observed that a 25 % loss of growth rate is observable only at concentrations of ethanol above 20 g/L. In our experiments, no more than 5 g/L can be found even in the batch S. cerevisiae axenic cultivations (Figure 2); therefore, we expected this inhibition parameter to be sufficiently small to be not taken into account and even less relevant during continuous cultures. However, the model can consider inhibitions described in the supplementary model definition (Supplementary file S1_file, equation 9). However, fitting solutions to increase the model’s fitness to the continuous culture rendered the effect of this inhibition non-relevant, as expected. Despite this, we acknowledge that further optimizations could be done in the model inhibition to account for more precise growth and metabolic inhibition data but would probably require individual pathway inhibition constants. Therefore, more data would be needed to fit them. However, we believe this increase will be more relevant as the complexity of the model increases to address other more intricate relationships between strains in the future.*
3. Figure 4A – What is meant by fraction of events?
   a. *It refers to the proportion of FC events for each strain regarding the total FC events. Each event represents a single cell after the cleaning procedures described on the material and methods. To increase the clarity of the document and the figure, the term “fraction of events” has been changed to “FC events fraction.”*
4. Figure 4A & B – It seems a little odd that E. coli psi values are constant throughout the simulations. I would have imagined these values to show a trend similar to Figure 4C [GLUferm].
   a. *Figure 4 A refers to the proportion of cells found in the fermenter, measured in terms of the “FC events fraction”, and their transition constants should be in the order of duplication times and dilution rates. Therefore, we would not expect them to behave within the same dynamic range as Figures 4 A and B, which depict the value of the commitment of enzymatic resources of each strain towards each specific metabolic state in terms of the “Relative enzyme fraction” as described in equations 2 to 5. On the other hand, for figure 4 B and C, the dynamic stability depends on the interplay of the rate of change of this “Relative enzyme” as established in equation 4, and the contribution of each metabolic state on the total metabolic cell objective (growth rate, in this work). Figure 4 B refers to E.coli in which the growth rates provided by GLU consumption are significantly higher than the other two considered metabolites (ACE and ETH), resulting in a very tight restriction to change the enzymatic commitment in E.coli. In other words, the considerable difference in growth rates even at small GLU concentrations represses the increase of usage of other substrates, as expected from the known GLU catabolic repression in this strain. The use of cybernetic models to render these regulatory effects resulting from the competition or fitness of different metabolic options (substrates) has been previously described and discussed in the works of Kompala, Ramkrishna, and collaborators [24,30,31,70,71].*
5. Figure 4C- it is very hard to see the trend for ACE. Suggest the authors to plot these values as separate graph.
   a. *We are very sorry, but we have tried many configurations for this figure, even 3D variations and this is the best we have been able to find to present the comparison of behaviour of all metabolic pathways.*

Fluctuating environmental conditions provide periodic fitness advantage and can lead to population stability in continuous cultures

Lines 354-374, Figures 6A, 6B

1. The authors conclude from Figure 6A that for a few values of D [ranging from 0.025-0.1 h-1], the coculture is stable. However, looking at the figures, it seems to me that E. coli is growing at the cost of S. cerevisiae at D >= 0.075 h-1, similarly the converse for D <0.075h-1 and that the co-culture is not ‘stable’ per se.

a. *Thanks for your observation. In effect, in some cases of all simulations above D= 0.075h-1 E.coli is growing and dominating the culture against S.cerevisiae, and conversely, in some of the solutions found below D=0.075h-1 S.cerevisiae have growth accumulations higher than E.coli. Each of the plots is comprising 231 simulations with different frequencies and feed step times with different degrees of stability in terms of the rates at which the ratio between the different population change as the continuous culture is prolonged. In lines now 376-378 we approach this by stating that the simulations show features “leading to potential co-culture stabilization”, and not concluding firmly on the exact term of them being “stable”.* *To increase clarity, we changed to “leading to the potential conservation of the species in the co-culture”.*

2. Figure 6B & Lines 354-374 – The Figure 6B shows all the growth rate simulations at w = 0.1, s= 0.1 and are discordant with Lines 364-366.

a. *We are very sorry for the discrepancy; Figure 6B has been corrected to show the real values of w and s on top of each simulation.*
4. Line 365 – Fitness advantage – How do the authors define fitness advantage? Is it the ability of the organism to grow at its’ maximum capacity?
   a. *In this work, corresponds to the ratio between the effective growth rate achieved in the environment and the maximum growth rate. The latter definition is also used as the metabolic objective defining each strain’s fitness for each metabolic pathway option. Therefore, the expanded concept of fitness advantage is defined as the ability of the organism to grow at higher speeds than its competitors in the co-culture.*

Population stability resulting from intermittent feeding is frequency

1. Figure 7A – What is meant by fraction of events?
   a. *As previously stated, It is referring to the proportion of FC events for each strain regarding the total FC events, and the term has been changed to “FC events fraction”*
2. Lines 394-398 – Suggest the authors to include interaction term (as above).
   a. *Thank you for your suggestion. Please see our answer above.*

3. Figure 7F – Same comments as Figure 4C.

a. *As previously discussed, we wouldn’t expect the same dynamics between the two strains due to the different values of maximum consumption rates, yields, and saturation constants.*

Minor comments

2. Page14 – Line 355 – What are w and s parameters? Please mention in the main text.
   a. *The definition of w and s has been extended from Fig 5. To the text at line (351) by “Pulse profiles were defined by square waves with dilution rates (D), frequencies (w) and pulse duration fraction (s).” and to the nomenclature box.*

Co-culture dynamics involves the active phenotypic diversification of the yeast population

Authors carry out experiments with GFP tagged S. cerevisiae and seek to show that there is a variation in phenotype. Following points are less clear to me:

1. How can the same set of parameters determined with wild type S. cerevisiae be used for GFP tagged S. cerevisiae? I would imagine these to be different for both.
   a. *While the GFP tag production is indeed presenting a metabolic burden to the cell, their production is not set into a constitutive strong promoter but a stress glc3 promoter and included only once in the chromosome. Furthermore, the tag does not interfere with the stress response by glc3 operon. We have found no significant reduction in the maximum growth rate of this strain; therefore, we expect the effect of the tag to be small for the purposes of this work.*
2. Figure 9 – There are two (C), please modify the names.
   a. *We* are *sorry for the subfigure labelling mistake; please be sure it has been corrected.*
3. Line 496 – Line 501 – Without E. coli data, it is hard to conclude Lines 498-501.
   a. *It’s true that the phenotypic diversification of the E. coli population has not been tracked. However, our previous data have shown that yeast is more prone to exhibit diauxic shift than E. coli, and that’s why we focused our effort on the characterization of the stress response of yeast cells. Also, it is expected to observe a higher stress response for the yeast population under these cultivation conditions since they can be easily outcompeted by E. coli which is a fast grower. However, we can understand the concern of the reviewer and we have reformulated the sentence in order to avoid overstatement “ Taken altogether, the data pointed out that co-culture stability could be ensured by active diversification of the yeast population giving it more metabolic flexibility and fitness under fluctuating environmental conditions”.*

- In supplementary data file 2, the authors provide details on the simulations however this file needs to be updated with the correct information and links so that the readers can access MONKS, this is currently not available and there are placeholder symbols where links should be.

a. *Thanks for the observations. The database repository and the GitLab addresses can be found on lines now 130 and 131, and 196 on the main text. We are very sorry for the omission of these data on the supplementary data file 2, which has been corrected accordingly.*

After addressing the assessments, we have also reviewed the resolution of all figures enhanced for better display after file conversion to PDF. Furthermore, we reviewed all references and the disponibility of all datasets and software toolboxes as well as their documentation.

Thank you for considering our manuscript.

Sincerely,
